# Evaluating the effectiveness of telehealth-delivered Child and Family Traumatic Stress Intervention (CFTSI) for children and adolescents following recent trauma: a multi-site randomized controlled trial in France

**DOI:** 10.1186/s40359-025-03585-0

**Published:** 2025-11-12

**Authors:** Erica Fongaro, Diane Purper-Ouakil, Marie-Christine Picot, Carrie Epstein, Hala Kerbage

**Affiliations:** 1 Montpellier University Hospital, Department of Child and Adolescent Psychological Medicine, Montpellier, Hérault France; 2 INSERM U 1018, CESP, Developmental Psychiatry/ADHD and emotional disorders, Villejuif, France; 3 Department of Biostatistics and Epidemiology, University Hospital, Montpellier, France; 4 Yale School of Medicine Child Study Center, 230 South Frontage Road, New Haven, CT 06520 USA

**Keywords:** Post-traumatic stress, PTSD, Early intervention, Trauma, Family therapy

## Abstract

**Background:**

Children and adolescents exposed to traumatic events (TEs) are at risk of developing post-traumatic stress disorder (PTSD), with long-term emotional, cognitive, and social consequences. Early interventions within the first months post-trauma are essential, yet structured, evidence-based approaches remain limited. The Child and Family Traumatic Stress Intervention (CFTSI) is a brief, family-centered approach that enhances parental support and reduces post-traumatic symptoms. While effective in the U.S., its implementation in European settings, particularly via telehealth, remains unexplored.

**Objective:**

This study evaluates the effectiveness of telehealth-delivered CFTSI in reducing post-traumatic symptoms in children aged 7–17 after recent TEs, compared to non-specific psychological support. Secondary objectives include assessing symptom persistence at three months, parental PTSD symptoms, and predictors of treatment response.

**Methods:**

This study is a multicenter, evaluator-blinded RCT comparing CFTSI to supportive counseling. Eligible children (aged 7–17) must have experienced a TE within the past 45 days and exhibit at least one posttraumatic stress symptom. Exclusion criteria include severe cognitive impairment, ongoing abuse, or acute psychiatric emergencies.The CFTSI group will receive 5–8 structured, family-focused telehealth sessions targeting parent-child communication and coping. The control group will receive supportive counseling. The primary outcome is post-traumatic symptom reduction, assessed using the Child Posttraumatic Stress Checklist 3 months after completion of the program. Secondary outcomes include three-month symptom persistence, parental PTSD symptoms, and predictors of response. Analyses will use ANCOVA and regression models adjusted for baseline characteristics.

**Discussion:**

This is the first RCT in France evaluating telehealth-delivered CFTSI. Conducted across four sites, findings will inform trauma care integration at regional and national levels. By improving accessibility and supporting trauma-exposed families, this study aims to enhance clinical training and advance evidence-based early interventions.

**Trial registration:**

Clinicaltrials.gov. Number NCT07031817. Registered on the 2nd of October, 2025.

## Background

Children and adolescents worldwide are exposed to alarmingly high rates of sexual abuse, physical assault, violent accidents, and other potentially traumatic events (TEs) [1–3], as defined by the DSM-5 criteria [4]. Childhood exposure to trauma is associated with an increased risk of mental health disorders, including post-traumatic stress disorder (PTSD), depression, anxiety, and substance use disorders, with potentially lasting effects on development and well-being [5, 6]. Traumatic experiences can also disrupt family dynamics, affecting not only children but also their parents, who may develop PTSD symptoms themselves [7].

Although some children experience spontaneous recovery following a single TE, PTSD remains prevalent, with 11% of children aged 5–18 years still meeting diagnostic criteria one year after the traumatic exposure [8]. PTSD in children and adolescents has well-documented negative effects on social, emotional, educational, and developmental outcomes [9]. While some individuals eventually recover, acute symptoms—such as intrusive thoughts, hyperarousal, irritability, fearfulness, sleep disturbances, and concentration difficulties—can cause significant distress for both the child and their family. These symptoms not only result in immediate suffering but also contribute to functional impairment and may hinder optimal development [10]. The persistence and severity of posttraumatic symptoms are influenced by multiple factors, including the type of TE, parental mental health, and the availability of family and social support [3].

Although multiple evidence-based treatments exist for PTSD in children [11], there is limited research on early interventions—those delivered within the first months of trauma exposure—to alleviate acute post-traumatic symptoms and strengthen family support, potentially playing a role in preventing chronic posttraumatic symptoms. The Child and Family Traumatic Stress Intervention (CFTSI) is a brief, evidence-based early intervention developed at the Yale Child Study Center for children aged 7–17 who have recently experienced a TE [12, 13]. This family-centered intervention consists of 5–8 structured sessions designed to enhance parental support, improve parent-child communication, and teach targeted coping strategies to reduce acute post-traumatic symptoms. CFTSI has been implemented in children exposed to various TEs, including sexual and physical abuse, domestic and community violence, road accidents, and injuries [13, 14]. A randomized control trial (RCT) by Berkowitz et al.. found that children receiving CFTSI had significantly fewer full and partial PTSD diagnoses at three months compared to a control group receiving psychoeducation and non-specific psychological support [13]. Additionally, a multicenter study involving 1,190 families demonstrated that CFTSI significantly reduced acute post-traumatic symptoms, regardless of age, gender, trauma type, or history of prior exposure [14]. A meta-analysis further supported its effectiveness, showing reductions in both child and parent PTSD symptoms, aligning with extensive literature on the impact of parental mental health on child recovery following trauma [15]. Parents experiencing distress or post-traumatic stress in response to their child’s trauma may struggle to provide emotional stability, thereby exacerbating the child’s difficulties [16]. Conversely, when parents receive adequate support and demonstrate effective coping strategies, children exhibit better adjustment, greater self-efficacy, and improved emotional regulation [3, 14]. Strengthening parental capacities after trauma is therefore essential for mitigating long-term consequences and fostering resilience. Additionally, recent research has also explored an online adaptation of CFTSI, demonstrating its feasibility and effectiveness in reducing PTSD symptoms in both children and parents, including those with repeated trauma exposure [17].

At the posttraumatic stress unit in the Child and Adolescent Psychiatry Department in the University Hospital of Montpellier (France), we have begun implementing CFTSI in routine clinical practice following training with the founders of the CFTSI. However, despite its demonstrated efficacy in the U.S., no RCTs have been conducted in France or other countries to evaluate CFTSI’s effectiveness in reducing acute symptoms in children and parents and its impact at three months post intervention. Moreover, a qualitative study exploring the experiences and perceived needs of parents of adolescents aged 11 to 17 who had been exposed to a TE within the past three months in Montpellier revealed that parents struggled to understand their children’s reactions, lacked explanations and knowledge about post-traumatic stress, and expressed feelings of failure and parental inefficacy in the face of their child’s suffering [7]. They were uncertain about the best approach to take in response to conflicting needs and tended to alternate between avoidant parenting strategies and coercive parental behaviors. This, in turn, affected their relationship with their child and led to conflictual dynamics [7]. These qualitative data highlight the need to provide parents with an early, adapted, and validated family intervention following their child’s traumatic exposure.

A family-centered approach such as CFTSI not only supports the child but also addresses parental distress, which is crucial for a stable recovery environment. The online format of CFTSI may enhance accessibility, reduce stigma, and expand the reach of trauma care. Given the lack of validated first-line early posttraumatic interventions for children and adolescents, testing CFTSI’s effectiveness over standard psychological support in a French population is a critical step towards integrating it as a standard care option at regional and national levels. Furthermore, a RCT involving the telehealth delivery of CFTSI may offer a practical, scalable solution to facilitate child and family recovery after traumatic exposure. This paper presents the study protocol for the RCT being conducted across four sites in France to examine the effectiveness of the CFTSI in reducing posttraumatic symptoms in children and their parents following the telehealth delivery of the intervention and at 3 months follow up.

## Methods

### Aims of the study

The primary aim of the RCT is to assess the effectiveness of the CFTSI in reducing post-traumatic symptoms measured by the validated French version of the Child Posttraumatic Stress Checklist [18] (CPC) at 3 months after completion of the program, in children and adolescents aged 7 to 17, exposed to a TE within the last 45 days, compared to standard child-centered psychological support.

The secondary aims of the study are the following:

• To examine the effectiveness of the CFTSI in reducing post-traumatic symptoms in children and adolescents on the CPC, at 3 months after completion of the program, compared to standard child-centered psychological support.
• To examine the effectiveness of the CFTSI in reducing post-traumatic symptoms in parents on the validated French version of the Posttraumatic Stress Disorder Checklist for DSM-5 (PCL-5) [19], immediately after completion of the program and at three months follow-up, compared to standard child-centered psychological support.
• To investigate factors associated with the child post-traumatic symptom scores on the CPC prior to any intervention (across all included participants):
  - ◦ Gender, age, and socioeconomic status.
  - ◦ Prior traumatic exposures measured by the adapted French version of the CPC.
  - ◦ The presence of pre-existing psychiatric or neurodevelopmental disorders measured by the validated French version of the Schedule for Affective Disorders and Schizophrenia for school-age children, Kiddie-SADS, lifetime child and parent versions, K-SADS [20].
  - ◦ The presence of one or multiple traumatic exposures in the parent, assessed using the Life Event Checklist (LEC-5) [21].
  - ◦ A lower score on the resilience measurement scale assessing social and family support and the child’s skills: Child Youth Resilience Measure (CYRM), child version (one version for ages 7–10 and another for ages 11–17) [22, 23].
  - ◦ The severity of posttraumatic reactions among parents measured by the PCL-5.
  - ◦ The type of TE.
• To investigate factors associated with changes in the CPC scores post-CFTSI:
  - ◦ Gender, age, socioeconomic status.
  - ◦ Prior traumatic exposures measured by the adapted French version of the CPC.
  - ◦ The presence of pre-existing psychiatric or neurodevelopmental disorders measured by the K-SADS [20].
  - ◦ Changes in parent post-traumatic symptoms at the PCL-5 post CFTSI.
  - ◦ The presence of one or multiple traumatic exposures in the parent, assessed using the LEC-5 [21].
  - ◦ The type of TE.
  - ◦ The timing of the CFTSI delivery (for example, initiation of CFTSI within 60 days following the TE versus 30 days).
  - ◦ The evolution of the score on the resilience measurement scale assessing social and family support and the child’s skills: CYRM, child version (one version for ages 7–10 and another for ages 11–17) at baseline, immediately after completion of the program and at 3 months after the completion of the program [22, 23].
•To examine the concordance between child and parent-reported post-traumatic symptoms by comparing the child version and the parent version of the CPC at baseline, immediately after completion of the program, and 3 months after completion of the program.
• Examine the concordance between the perception of social support and the child’s skills as reported by the child and by the parents, at baseline, immediately after completion of the program, and 3 months after completion of the programby comparing the scores on the child and parent versions of the CYRM.

### Trial design

The study is a single-blind effectiveness multi-site superiority RCT, comparing the telehealth delivery of the CFTSI to the online delivery of child-centred psychological support with two parallel arms and blinded assessments of the outcome measures. Outcomes on child and caregiver measures will be assessed at baseline (T0), immediately after completion of the program (T1), and 3 months after completion of the program (T2). The primary outcome point is set as T1. The Standard Protocol Items: Recommendations for Interventional Trials (SPIRIT) [24] is outlined in Table 1.

**Table 1:** Schedule of enrollment, interventions and assesments of primary and secondary endpoints; V = visit; T = time

Visit (V)	Pre-inclusion V0 Screening T −1 to 2 weeks	Inclusion V V1 Baseline T0	Psychological Intervention 1 + 5 weeks (1 session/week for 5 weeks) ± 1 to 3 additional sessions as needed over three weeks (2 months)	Follow-up V V2 T1(immediately after completion of the program ± 7 days)	Follow-up V V3 T2 days (3 months after completion of the programs ± 7 days)
*Date*		*Day 1*		*Day 35*	*Day 185*
Protocol explanation	✓				
Oral consent		✓			
Randomization		✓			
K-SADS parents		✓			
K-SADS children		✓			
Socio-demographic questionnaire		✓			
PCL-5		✓		✓	✓
LEC-5		✓			
CPC child version		✓		✓	✓
CPC parent version		✓		✓	✓
CYRM child version		✓		✓	✓
CYRM parent version		✓		✓	✓
MFQ child version			Session 2 of the CFTSI group		
MFQ parent version			Session 1 of the CFTSI group		
PTSD-RI child version			Session 2 of the CFTSI group		
PTSD-RI parent version			Session 1 of the CFTSI group		
Family booklet			Sessions 3, 4, and 5 of the CFTSI group		
MFQ common version			Sessions 4 and 5 of the CFTSI groupCFTSI		
PTSD-RI common version			Sessions 4 and 5 of the CFTSI group CFTSI		
Developmental history			Session 1 of the CFTSI group		
THQ adapted adult and parent version			Session 1 of the CFTSI group		
THQ adapted child version			Session 2 of the CFTSI		
PCL-C *PCL-5 adapted to CFTSI			Session 1 of the CFTSI group		
Satisfaction questionnaire			Session 5 of the CFTSI group		

### Study setting

The recruitment will take place among parents seeking consultation for their children at one of the four branches of the Regional Center for Posttraumatic Stress in the Region of Occitanie in France, within 45 days of the child’s exposure to a TE. The Regional Centers for Posttraumatic Stress in France provide a comprehensive care system—including evaluation, orientation, and treatment—for individuals suffering from PTSD across all ages. These centers are affiliated with hospitals and cover all regions of France. The center in the region of Occitanie serves four cities: Montpellier, Toulouse, Nîmes, and Thuir. Both interventions (CFTSI and child centered supportive psychotherapy) will be delivered by psychologists at the University Hospital of Montpellier. Clinicians delivering the CFTSI will have received prior CFTSI training and supervision by the founders of the CFTSI. The interventions will be conducted entirely online. This approach will facilitate participation for families from centers outside Montpellier and enhance parental compliance, particularly for those facing time or transportation constraints. Recruitment will begin in December, 2025 and is expected to be completed by June 2027.

## Participants

### Eligibility criteria

Families will be included if they meet the following inclusion criteria: (i) have a child or adolescent aged 7 to 17 years living in the family home, (ii) the child has been exposed to a TE aligning with the DSM-5 criteria for TEs within the past 45 days, (iii) there is at least one symptom on the Child Post-Traumatic Stress Checklist (CPC) at T0, (iv) at least one of the parents is available to participate in the intervention, (v) being fluent in French, both orally and in writing, and (vi) no concurrent participation in other therapeutic interventions aimed at treating traumatic stress symptoms during the inclusion period.

Participants will be excluded if they meet any of the following criteria: (i) parents and/or children present an imminent risk requiring immediate case management at the time of recruitment, or severe psychiatric disturbance or risks requiring specialist mental health services. This will be assessed by a clinical evaluation while gathering consent (preinclusion) and will be based on the clinical judgment; (ii) children are in foster care or in the process of being placed; (iii) there is suspicion of maltreatment or abuse perpetrated by one or both parents; (iv) children and/or parents have a known and/or documented intellectual disability, and (v) there is an absence of informed consent from at least one parent and/or refusal of participation by the child.

### Interventions

#### The CFTSI

The CFTSI intervention consists of five to eight structured sessions, each lasting approximately 1 h, delivered at a pace of one session per week. Psychotherapists trained in CFTSI will implement the intervention solely in the experimental group, following its standardized protocol. The intervention includes Session 1, parent(s) alone; Session 2, child alone, Sessions 3–5, conjoint sessions with the child and parent(s). Additionally, one to three supplementary sessions may be conducted as needed to address specific points or review coping strategies in greater detail. The CFTSI is designed to enhance symptom recognition, improve parent-child communication, and teach targeted coping skills [10, 13–15]. The intervention focuses on key post-traumatic reaction clusters, including sleep disturbances, social withdrawal, intrusive thoughts, anxiety, anger outbursts, oppositional or aggressive behaviors, and trauma induced alterations in cognitions (e.g., guilt). A detailed CFTSI manual outlines the structure of each session, including standardized questionnaires used to assess symptom intensity, frequency, and progression over time. These questionnaires serve a dual function: they facilitate psychoeducation and improve communication around symptoms, helping both children and parents to better identify and articulate post-traumatic experiences. The CFTSI-specific questionnaires and family agenda, which records strategies learned during sessions, have been translated into French by our team. These questionnaires are fully integrated into the clinical interview and administered by clinicians within CFTSI sessions. They do not require additional effort from participants nor extra time outside of the sessions, except for the family log, which is introduced in session 3. Several questionnaires throughout the sessions serve as discussion tools. However, these are adapted clinical versions distinct from those used in blinded assessments for outcome measures. These adaptations, developed by the founders of CFTSI, have been translated into French with their consent.

#### Fidelity

To evaluate fidelity of the CFTSI to the treatment model, clinicians will complete a session checklist at each session to indicate which components were completed, which family members attended, and whether any rescheduling was needed. All sessions will be recorded and 20% of the recordings will be reviewed by a supervisor who is trained in CFTSI, who will complete a structured observation form, developed for the CFTSI, to score which elements of the intervention have been carried out by the clinician and to what quality. Additionally, regular supervision and coaching sessions will take place with experienced and trained CFTSI supervisors of the Yale Child Study Center.

### Control group

The control group will receive five to eight weekly sessions of unstructured child-focused psychological supportive therapy conducted online. This form of supportive therapy is already part of the standard care practice at the four sites of the Occitanie regional centers for posttraumatic stress and aims to provide emotional support while strengthening the child’s daily adaptation skills. It will be delivered by psychotherapists from the Montpellier University Hospital who are not trained in CFTSI. This ensures a consistent online format, allowing for better comparability between the intervention and control groups.

### Informed consent and assent (pre-inclusion)

During the pre-inclusion visit, the investigator provides detailed information to the parent(s) and child, addressing any questions about the study’s objectives, potential constraints, foreseeable risks, and expected benefits. The investigator also explains the rights of research participants and verifies the eligibility criteria. Following this discussion, the investigator provides the parent(s) with a copy of the information sheet. The child also receives age-appropriate informational materials (for ages 7–11 or 12–17) and is informed about the study procedures. The child’s personal assent is sought, and their decision is fully respected. If the child chooses not to participate, they will not be included in the study. After this information session, parent(s) are given a 3 days reflection period to make an informed decision, in order to keep the intervention as soon as possible. If they choose to participate, they give their consent orally to the investigator, which is obligatory before any clinical or paraclinical assessments related to the study are conducted. An attestation will be signed by the investigator to document these oral agreements, collected in the e-CRF for traceability and the participation will be recorded in the patient’s medical file.

### Outcomes

We will aim to start intervention sessions no more than 2 weeks after T0 assessments, and to conduct T1 assessments on the day of the last session of the intervention. Interventions in both groups will last a maximum of 8 weeks. T2 assessments will be scheduled at 12 weeks following T1 (approximately 20 weeks after T0). In case participants do not attend a scheduled assessment, three attempts will be made to contact them in the same week, and once the following week, to schedule a new appointment. This will be done via phone calls. Interviews will be carried out on site or online by trained master-level or doctorate-level clinicians. Investigators involved in assessments will be blinded as regards program/treatment status.

#### Primary outcome

The CPC (child version) is the primary outcome measure of the study [18]. This scale assesses post-traumatic stress symptoms in children aged 7 to 18 based on DSM-5 criteria [25]. It includes 21 items evaluating symptom frequency (e.g., intrusion, avoidance, hyperarousal) and 6 items assessing functional impairment. Responses are rated on a Likert scale from 0 (never) to 4 (daily), with higher scores indicating greater symptom severity. The scale has been validated in French with robust psychometric properties. Clinicians, blinded to group allocation, will administer the CPC to ensure consistency and reliability [26]. Time Frame: Baseline, immediately after completion of the program, and 3 months after completion of the program.

#### Secondary outcomes

– Evolution by the variation in the total symptom score on CPC of post-traumatic stress symptoms in children at baseline, immediately after completion of the program, and 3 months after completion of the program.
– Evolution of post-traumatic stress symptoms in parents, at baseline, immediately after completion of the program, and 3 months after completion of the program using the Post-Traumatic Stress Disorder Checklist for DSM-5 (PCL-5). The PCL-5 is used to evaluate PTSD symptoms in parents [27]. It includes 20 items corresponding to DSM-5 diagnostic criteria, with scores ranging from 0 (no symptoms) to 80 (severe symptoms). Subscales assess intrusion, avoidance, cognitive and mood alterations, and hyperarousal. It has been validated in French with good psychometric properties. In the PCL-5, a score of 2 or more on any item (scale: 0 to 4) is considered clinically significant. The score can range from 0 (no symptoms) to 80 (maximum symptoms).
– Correlation at baseline between CPC total score (child version) and variables from a socio-demographic and developmental questionnaire.
– Correlation at baseline between CPC total score and presence of a pre-existing psychiatric or neurodevelopmental disorder prior to the TE assessed by the K-SADS.
–Correlation at baseline between the CPC total score and trauma history (number of prior TEs) and parental trauma (LEC-5). The presence of one or multiple past traumatic exposures in one or both parents, will be assessed using the Life Event Checklist-5. The Life Event Checklist DSM-5 version [21] consists of a list of 16 potentially TEs based on Criterion A of PTSD in the DSM-5. For each event, the participant must indicate their level of exposure (experienced directly, witnessed, learned about, or work-related).
– Correlation at baseline between CPC total score and parental PCL-5.
– Correlation at baseline, immediately after completion of the program, and 3 months after completion of the program between CPC total score and CYRM-28 child version. Protective factors such as social and family support and the child’s skills are assessed using CYRM (one version for ages 7–10 and another for ages 11–17). The CYRM [22] is a globally used scale for measuring social and family support and the child’s skills. It was translated and validated in French by a Canadian team [23] and consists of 17 items assessing social and family support and the child/adolescent’s skills in daily life. The child version has two forms: one for ages 7–10 and another for ages 11–18. There is also a parent-report version that assesses their perception of the child’s social and family support and skills (one for ages 7–10 and another for ages 11–18).
– Correlation between CPC total score and parental PTSD symptoms (PCL-5) at baseline, immediately after completion of the program, and 3 months after completion of the program.
– Correlation between Child Posttraumatic Stress Checklist (CPC) total score and parental presence during the program period (weekly parental presence, 1 session per week, up to 8 weeks)]
– Correlation between Child Posttraumatic Stress Checklist (CPC) total score and time elapsed before Child and Family Traumatic Stress Intervention (CFTSI) implementation. The delay before CFTSI implementation is the time, in days, between index trauma and the first CFTSI session.
– Mean change in PTSD symptoms (PTSD-Rating Index) during the program period (weekly, up to 8 weeks). PTSD symptoms assessed using the PTSD-RI, clinician-rated, during each CFTSI session. Score ranges from 0 to 88: higher scores indicate more severe symptoms. This questionnaire, administered by clinicians, is integrated into CFTSI sessions and is used in each session as a tool to enhance symptom identification and communication.
– Mean change during the program period (weekly up to 8 weeks) in MFQ score. MFQ score ranges from 0 to 66 (higher scores indicate more severe depressive symptoms). This questionnaire, administered by clinicians, is integrated into CFTSI sessions and is used in each session as a tool to enhance symptom identification and communication.
– Concordance at baseline, immediately after completion of the program, and 3 months after completion of the program between child- and parent-reported post-traumatic symptoms via CPC child and parent version.
– Concordance between child- and parent-reported child’s perception of social support and skills via CYRM child and parent version at baseline, immediately after completion of the program, and 3 months after completion of the program.

#### Other assessments

A detailed socio-demographic and early development questionnaire will be used to collect information about the child’s age, gender, family structure, socio-economic status, and developmental milestones. It will also include questions on prenatal, perinatal, and early childhood history, as well as previous medical or psychological interventions. The CPC will be used to evaluate the child’s history of exposure to TE, including physical and sexual abuse, natural disasters, accidents, and other adverse experiences. The questionnaire includes versions adapted for children and parents, ensuring comprehensive data collection. The K-SADS, a semi-structured diagnostic interview, will be used to assess pre-existing psychiatric or neurodevelopmental disorders in children. This tool is validated in French and will be administered at baseline to confirm eligibility and gather diagnostic data [20, 28]. The THQ [29], adapted by the founders of the CFTSI for adult use (assessing the parent’s own history of traumatic exposure) and for use by parents (assessing the child’s history of traumatic exposure as reported by the parent), is not the same version used for the secondary outcome measure. Instead, we used the clinically adapted version developed by the CFTSI founders, which we translated ourselves.

#### Intervention adherence and fidelity

Adherence will be monitored by tracking the number of sessions attended by participants in both the CFTSI and control groups. Completion of all five core sessions will be considered full adherence, while drop-outs will be documented for families attending fewer sessions. To monitor fidelity, all sessions will be recorded, and a sample of 20% of the recorded sessions will be reviewed and assessed by a supervisor trained in CFTSI, who will complete a structured observation form, developed for the CFTSI, to score which elements of the intervention have been carried out by the clinician and to what quality. Additionally, regular supervision and coaching sessions will take place with experienced and trained CFTSI supervisors of the Yale Child Study Center.

#### Family impact

Observations from joint sessions in the CFTSI group will provide qualitative data on parent-child interactions and the family’s ability to communicate about trauma-related symptoms. These interactions will be analyzed to evaluate changes in family dynamics over the course of the intervention.

#### Satisfaction questionnaire

At the end of the intervention (session 5 for the CFTSI group), parents and children will complete a satisfaction questionnaire to assess their perceptions of the intervention’s effectiveness and their overall experience.

### Randomization

At the inclusion visit, randomization will be conducted after verifying eligibility criteria and having obtained informed consent. The investigator will perform the randomization using the e-CRF system via Ennov Clinical^®^ software. Once the randomization is completed, the system will immediately generate the participant’s unique study identification number and assign them to either the control or experimental group. The site will then notify the investigator of the randomization result and group allocation.

### Sample size

According to Hahn’s (2019) [15] meta-analysis, the effect size (Hedge’s g) in favor of the CFTSI intervention ranged from 0.86 to 1.17 (*N* = 640 children). In a Cochrane 2016 meta-analysis, comparing all psychotherapies versus control (short-term standard care), on total PTSD symptoms, the effect size is −0.53 (CI95%: −0.94; −0.12) when looking at family therapies [30].

Taking this effect size of −0.53 with a power of 80% and a two-sided alpha risk of 5%, it is necessary to include 57 patients per group (NQuery software). Given an expected drop-out rate of around 20%, it is necessary to include 70 patients per group, i.e. 140 patients in total.

### Blinding

Although this is an open-label study, the investigators responsible for conducting assessments will remain independent of the intervention process and will not be involved in delivering the therapy. To minimize bias in outcome evaluation, parents and children will be reminded before each assessment not to disclose details regarding the type of intervention they received. Since this study evaluates non-pharmacological interventions, there is no procedure for unblinding a participant’s allocated intervention. Given the nature of the study, it was deemed unnecessary to establish specific unblinding circumstances or protocols.

### Data collection methods

All assessments will be collected for each participant in accordance with the e-CRF developed for the study. Data will be recorded by the investigator(s) or clinical study technician(s) involved in the trial, following the standardized procedures outlined in the protocol. Real-time quality control will be ensured through the use of the Ennov Clinical^®^ software platform, which complies with regulatory standards for clinical data management.

### Data management

The study data are entered into the e-CRF developed from the ENNOV CLINICAL software, which allows real-time quality control of the data. In order to meet regulatory requirements, this software complies with FDA recommendations concerning Computerised Clinical Trial Management Systems and electronic signatures, as well as standards (CDISC, ICH, GCP 2001/20/EC, etc.). Connection to the eCRF is via a password and a unique identifier for each user, which will only give access to patient data. An audit function is built into the software, enabling traceability of the data collected and any modifications made. The encrypted data will then be transmitted to the center responsible for data management via a secure internet connection. The e-CRF must contain all the information required by the protocol.

### Data processing

The individual data required to analyse the study must be:

– entered into the e-CRFs as they are obtained, whether they are clinical or para-clinical data.
– anonymised by the investigator.
– authenticated by an electronic signature from the investigator.
– all data are entered, which means that missing data must be justified.

Initially, the data entered into the e-CRF is checked and validated by the Clinical Research Monitor on the basis of the source documents.

Secondly, the data manager performs additional computerised consistency tests based on the presence of non-standard, missing, aberrant or inconsistent data, which are performed regularly during patient recruitment and follow-up. All consistency tests are defined in advance in a set of specifications proposed by the data manager and validated by the investigator, the statistician, the CRA monitor and the study TEC. Each inconsistency identified in the e-CRF (‘Query’) is the subject of a request for correction or justification to the investigator (response to the ‘Queries’). The investigator undertakes to make himself available to the members of the research team and to provide them with any additional information required to resolve these errors. This information is recorded in the study database.

Once the study has been completed, i.e. the data required by the protocol and any additional data entered (self-questionnaires), and the data monitored and validated, the study database is frozen.

The Clinical Research and Epidemiology Unit (URCE) at Montpellier University Hospital is responsible for maintaining the database. The data is stored in ASCII format.

### Data coding

Data coding is integrated into the e-CRFs. Drugs are coded when they are entered using their international non-proprietary name (WHO ATC database). Recoding of certain data is carried out prior to analysis by the study biostatistician.

### Data storage

Computer files and monitoring of any changes are backed up and made available by the Montpellier University Hospital CER. Server location: The application and database are hosted on the following servers OVH (Datacenter certified ISO 27001).

### Access protection

Authentication/Identification for access to the application:

Profiles define the functions and types of information accessible to users;

Logical access control is by password: minimum 8 characters blocked after three unsuccessful attempts;

Parameters defined in the CSAdministrator module.

### Database freeze

The database is frozen in accordance with the URCE (Clinical Research and Epidemiology Unit) procedures. The freeze is documented by a database freeze certificate and occurs only after all data have been verified and all corrections requested from the investigators have been completed.

### Statistical methods

The statistical analyses will be detailed in a statistical analysis plan (SAP) finalized before the last patient in the study is included.

#### Descriptive analysis

A flow chart describing participant numbers during follow-up will be provided. It will describe the number of patients included, randomized in each arm, exited in each arm, and analyzable for the main analysis. Discontinuation causes will be reported for each arm.

Patient characteristics will be described overall and by group for the ITT population. Quantitative variables will be described using standard statistics: mean and standard deviation, median and interquartile range, minimum and maximum values. Qualitative variables will be described by the numbers of each modality and the corresponding percentages.

#### Analysis of primary endpoint

The analysis of the primary endpoint will be performed on an intention-to-treat basis. The primary endpoint (pre-post intervention variation in the child version of the CPC score) will be compared between the two groups using an analysis of covariance (ANCOVA) in order to adjust for the stratification criteria of the randomization. The effect size will be determined using Cohen’s d.

#### Analysis of secondary endpoints

– Endpoints 1 (change in child CPC score between Baseline, immediately after completion of the program, and 3 months after completion of the program) and 2 (change in parent PCL-5 scores). The analysis will be carried out in the same way as for the primary endpoint.
– Endpoints 3 (relationship between different participant characteristics and pre-intervention CPC score) and 4 (relationship between different participant characteristics and response to treatment).
  The factors associated with the CPC score will be analyzed using multiple linear regression. Determinants of response to treatment (change in CPC score) will also be analyzed using multiple regression.

– Endpoint 5 (concordance between child and parent CPC scores).
  The concordance between the CPC score obtained by the child and that obtained by the parents will be analyzed at each measurement time using the intraclass correlation coefficient (ICC) and its 95% confidence interval.

– Endpoint 6 (concordance between child and parent CYRM scores).
  The concordance between the CYRM score obtained by the child and that obtained by the parents will be analyzed at each measurement time using the intraclass correlation coefficient (ICC) and its 95% confidence interval.

#### Sensitivity analysis

Sensitivity analyses will be carried out on the PP population to confirm the results obtained by the main analysis.

#### Exploratory analyses

### Observance

The number of performed sessions will be described. The discontinuation from the trial due to any cause (or due to specific causes) will be also reported and compared between groups.

Analyses for additional subgroups are not planned in the main study.

### Demographic and baseline characteristics

To ensure comparability, patient demographic and baseline characteristics will be summarized by group using descriptive statistics. For continuous variables, descriptive statistics (*n*, mean, standard deviation, standard error, median, minimum, and maximum) will be provided. For categorical variables, patient counts and percentages will be provided. Categories for missing data will be presented if necessary.

In the case of non-comparability for one or more parameters, an adjustment will be made on this/these parameter(s) for analysis of the judgment criteria. A multivariate linear regression model may be performed to account for potential confounders.

### Handling of missing data

In the presence of missing data, the mechanism for the appearance of this data will be studied for each of the variables. If the MNAR (Missing Not At Random) hypothesis is rejected, the M.C.A.R. (Missing Completely At Random) versus MAR (Missing At Random) hypothesis will be tested, by studying the relationship between the status of the variable (missing data or not) and the values taken by the covariates. Under the hypothesis of a MAR or MCAR mechanism, i.e. that the probability of non-response for a variable is independent of the value taken by this variable, a multiple imputation method [31] will be implemented. Analyses will be carried out independently on each of the complete databases and their results will be taken into account in estimating the final parameters and their standard deviations. Finally, the concordance of the results obtained between the complete database and the database after imputation will be studied. The main analysis will be based on the database with imputation of missing data in order to be as close as possible to the ITT population. The analyses will be carried out at the Clinical and Epidemiological Research Unit of the Montpellier University Hospital. All statistical tests will be two-sided and the significance threshold will be set at 5%. All statistical analyses will be carried out using SAS enterprise guide or R.

### Oversight and monitoring

An independent safety monitor will oversee compliance with regulatory requirements and verify data collection, ensuring adherence to the study protocol. Acting as a representative of the study sponsor, the monitor will ensure that all trial procedures meet regulatory and ethical standards.

Before the inclusion of the first participant, the monitor will conduct an initial site visit to present the study protocol and procedures. Investigators will receive detailed completion guidelines for the Case Report Forms (CRFs) to ensure consistency in data entry. Throughout the study, the monitor will have direct access to source documents necessary for verifying entries in the electronic Case Report Forms (e-CRFs) and other protocol-related records.

To maintain data integrity and reliability, the sponsor or its representatives will implement key measures, including providing instructional materials to study sites, conducting start-up training sessions for investigators and study coordinators, and ensuring proper completion of clinical report forms and study procedures. Regular site visits will be conducted, and ongoing communication with study personnel will be maintained via email, phone, or fax. Data from Case Report Forms will be systematically reviewed, and quality control checks will be performed on the study database to ensure accuracy and consistency.

### Harms

Any AE/adverse reactions/incidents related to the usual care of the patient will be declared by a health professional to the various circuits of sanitary vigilance applicable to each product or practice concerned (vigilance of the care, pharmacovigilance, materiovigilance, hemovigilance, cosmetovigilance, etc.) in conformity with the regulations in force.

### Auditing

An audit can be carried out at any time by people mandated by the sponsor and independent of the people carrying out the research. Its objective is to verify the security of participants and respect for their rights, compliance with applicable regulations and the reliability of data.

An inspection can also be carried out by a competent authority (ANSM for France or EMA as part of a European test for example). The audit, as well as the inspection, can be applied at all stages of research, from the development of the protocol to the publication of results and the classification of data used or produced within the framework of the research. Investigators agree to comply with the sponsor’s requirements for an audit and the competent authority for an inspection of the research.

### Confidentiality

In accordance with the legislative provisions in force, persons with direct access to the source data will take all necessary precautions to ensure the confidentiality of information relating to the investigational medicinal products, the research, the persons involved and in particular their identity and the results obtained. These persons, in the same way as the investigators themselves, are subject to professional secrecy. During the research or at its conclusion, the data collected on the persons involved and transmitted to the sponsor by the investigators (or any other specialist) will be made anonymous. Under no circumstances must the names or addresses of the persons concerned appear in clear text.

The sponsor will ensure that each person taking part in the research has given their oral consent for access to individual data concerning them and strictly necessary for the quality control of the research.

### Ancillary and post-trial care

Participation in the clinical-trial does not interrupt previous care in the participating centers, assuring that post-trial care is guaranteed.

## Dissemination plans

### Study report

Within one year after the completion or termination of the study, a final report will be prepared and signed by both the sponsor and the investigator. This report will be made available to the competent authority. The sponsor will also submit a summary of the final report to the CPP within the same one-year period following the end of the study.

### Publication

The publication of the main results will acknowledge the sponsor, all investigators who enrolled or monitored participants, as well as the methodologists, biostatisticians, and data managers involved in the study. It will also include the vigilance officers responsible for participant safety analysis and the members of any committees established for the research. The publication will comply with international authorship and publication standards (Uniform Requirements for Manuscripts, ICMJE, April 2010).

### Results communication

In accordance with Law No. 2002 − 303 of March 4, 2002, participants are informed, at their request, of the overall results of the research.

### Plans to give access to the full protocol, participant level-data, and statistical code

In accordance with the law n°2002 − 303 of the 4th march 2002, participants are informed, at their request, of general results of research by the investigator. For research purposes, the investigator can provide the full protocol, participant level-data, and statistical code on request.

## Discussion

This RCT evaluates the effectiveness of CFTSI compared to non-specific psychological support in reducing post-traumatic symptoms in children and adolescents aged 7–17 following exposure to a recent TE. The study also examines symptom persistence 3 months after completion of the program, the impact on parental PTSD symptoms, and the factors associated with baseline posttraumatic stress symptoms in children as well as the response to the CFTSI. Post-traumatic stress symptoms in children can lead to lasting emotional, cognitive, and social impairments. While evidence-based PTSD treatments exist, early interventions within the first three months post-trauma remain under-researched. CFTSI has shown promise in reducing post-traumatic symptoms and strengthening parent-child communication, yet its application in Europe remains limited.

This study is the first RCT in France to assess the effectiveness of the telehealth delivery of the CFTSI in reducing acute post-traumatic symptoms in children and parents across four sites in the region of Occitanie, in order to implement it at a regional and national level as a structured early intervention in routine care. The online format enhances accessibility, particularly for families facing logistical constraints.

Using validated tools such as the CPC and the PTSD Checklist for DSM-5 (PCL-5), this study ensures rigorous outcome assessment, while qualitative feedback will provide insights into acceptability and feasibility. It also assesses protective and resilience factors and their associations with outcomes and responses to the CFTSI. We expect CFTSI to significantly reduce post-traumatic symptoms, supporting its broader adoption as an early intervention model. This research will contribute to improving trauma care pathways, enhancing clinician training, and expanding accessible, family-centered psychological interventions for trauma-exposed children and their families.

## Data Availability

No datasets were generated or analysed during the current study.

## Abbreviations

ANSM: National Agency for the Safety of Medicines and Health Products
APA: American Psychiatric Association
CPC: Child Posttraumatic Checklist
CPP: Committee for the Protection of Persons
CFTSI: Child and Family Traumatic Stress Intervention
CRF: Case Report Form
CRPOcc: Occitanie Regional Psychotrauma Center
TE: Traumatic Event
AE: Adverse Event
GCP: Good Clinical Practice
ICH: International Council for Harmonisation
K-SADS: Schedule for Affective Disorders and Schizophrenia for school-age children
MESR: Ministry of Higher Education and Research
MFQ: Mood and Feelings Questionnaire
PCL-5: Posttraumatic CheckList
THQ: Trauma History Questionnaire
NDD: Neurodevelopmental Disorder
PTSD: Post-Traumatic Stress Disorder
RCT: Randomized Controlled Trial
